# The regulation of cellular metabolism by tumor suppressor p53

**DOI:** 10.1186/2045-3701-3-9

**Published:** 2013-02-06

**Authors:** Yingjian Liang, Juan Liu, Zhaohui Feng

**Affiliations:** 1 The Cancer Institute of New Jersey, University of Medicine and Dentistry of New Jersey, 195 Little Albany Street, New Brunswick, NJ, 08903, USA

**Keywords:** p53, Tumor suppressor, Cancer metabolism, The Warburg effect, Glycolysis, Oxidative phosphorylation, Lipid metabolism, Glutaminolysis, Antioxidant defense

## Abstract

As a hallmark of tumor cells, metabolic alterations play a critical role in tumor development and could be targeted for tumor therapy. Tumor suppressor p53 plays a central role in tumor prevention. As a transcription factor, p53 mainly exerts its function in tumor suppression through its transcriptional regulation of its target genes to initiate various cellular responses. Cell cycle arrest, apoptosis and senescence are most well-understood functions of p53, and are traditionally accepted as the major mechanisms for p53 in tumor suppression. Recent studies have revealed a novel function of p53 in regulation of cellular metabolism. p53 regulates mitochondrial oxidative phosphorylation, glycolysis, glutamine metabolism, lipid metabolism, and antioxidant defense. Through the regulation of these metabolic processes, p53 maintains the homeostasis of cellular metabolism and redox balance in cells, which contributes significantly to the role of p53 as a tumor suppressor. Further understanding of the role and molecular mechanism of p53 in cellular metabolism could lead to the identification of novel targets and development of novel strategies for tumor therapy.

## Introduction

Tumor suppressor p53 plays a central role in tumor prevention [1-4]. p53 is the most frequently-mutated gene in human tumors; DNA mutations in p53 occur in over 50% of all tumors and almost every type of tumor. Furthermore, it was estimated that p53 signaling is dysfunctional in over 80% of tumors through different mechanisms in addition to p53 mutations [1-5]. For instance, the negative regulators of p53, including MDM2, Pirh2, Cop1, and MDM4, are frequently amplified and/or overexpressed in many tumors, which leads to the dysfunction of p53 signaling [2,6-8]. In cervical cancer with a low mutation rate of p53, p53 is often inactivated and degraded by human papillomavirus E6 protein (HPV-E6) [9]. Disruption of normal p53 function is often a prerequisite for the initiation and/or progression of tumors. In mice, p53 knockout leads to the early development of various tumors, including lymphoma and sarcoma [10,11]. In human beings, Li–Fraumeni syndrome is a rare disease caused by the germline mutations of p53. Li–Fraumeni syndrome patients, who carry a germline heterozygous p53 gene, display a 50% cancer incidence by the age of 30 [12].

As a transcription factor, p53 mainly exerts its function in tumor suppression through transcriptional regulation of its target genes [1-4]. In response to a wide variety of intracellular and extracellular stress signals, including DNA damage, hypoxia, nutritional depletion and oncogene activation, p53 is activated primarily through posttranslational modifications, which leads to the increase of p53 protein half-life and therefore p53 protein accumulation in cells. The activated p53 protein then binds to a specific DNA sequence, termed the p53-responsive element, in its target genes to regulate their expression to start various cellular responses. Through these cellular responses, p53 facilitates DNA repair and inhibits the proliferation of cells that could potentially become cancerous. To date, over a hundred of p53 target genes have been identified [13]. Regulating cell cycle arrest, senescence and apoptosis are most well-understood functions of p53, which have been accepted as the main mechanisms for p53 to function as a tumor suppressor. Interestingly, recent studies have revealed that p53 regulates cellular energy metabolism [14-17], and antioxidant defense [18,19], which contribute greatly to the role of p53 in tumor suppression. This concept is supported by emerging evidence, including evidence from mouse models. For instance, a recent study showed that while p53 deficiency results in the elevated intracellular reactive oxygen species (ROS) levels, DNA oxidation and mutations in cells, dietary supplementation with antioxidant N-acetylcysteine substantially improves karyotype stability and prevents the early-onset tumors in p53 null mice [19]. In another most recent study, mice bearing lysine to arginine mutations at three (p53(3KR); K117R+K161R+K162R) of p53 acetylation sites were generated. p53(3KR/3KR) cells display impaired p53-mediated cell-cycle arrest, senescence and apoptosis. Unlike p53 null mice, which rapidly succumb to lymphomas, p53(3KR/3KR) mice did not develop early-onset lymphomas. Notably, p53(3KR/3KR) cells retain the ability to regulate energy metabolism and ROS production [20]. These results strongly suggest that unconventional activities of p53, such as metabolic regulation and antioxidant function, could be critical for tumor suppression.

### Metabolic alterations in cancer

The Warburg effect is the best characterized metabolic phenotype observed in tumor cells. In 1926, Otto Warburg found that unlike majority of normal cells which depends on mitochondrial oxidative phosphorylation to provide energy, most tumor cells primarily utilize glycolysis for their energy needs even under normal oxygen concentrations, a phenomenon termed “the Warburg effect” [21]. Compared with mitochondrial oxidative phosphorylation, which produces 36 ATP per glucose molecule, aerobic glycolysis is a much less efficient ATP-generating pathway, which only produces 2 ATP per glucose molecule. As a result, tumor cells have a much higher rate of glucose uptake and utilization than normal cells, and produce a large amount of lactate. Warburg originally hypothesized that tumor cells have a defect in mitochondrial oxidative phosphorylation which drives cells to rely on aerobic glycolysis for their energy needs. However, subsequent studies found that most tumor cells display a normal mitochondrial function, including normal capacity for mitochondrial oxidative phosphorylation. The biological significance of the Warburg effect in tumor development had been elusive for many decades since its discovery. It was unclear whether the Warburg effect contributes to tumorigenesis or was simply a by-product of tumorigenesis. Furthermore, the underlying mechanism for the Warburg effect was unclear. Nevertheless, the Warburg effect provides a base for Positron Emission Tomography imaging, which has been developed for tumor detecting since tumors take up more of the glucose analog ^18^flurodeoxyglucose than normal tissues. Remarkably, recent research has begun to answer these questions. These studies have led to the concept that the metabolic alterations are a hallmark of tumor cells [22-24]. Compelling evidence has shown that cancer cells depend upon metabolic alterations for continued growth, proliferation and survival [16,23-25]. To support the needs for the rapid growth and proliferation of cancer cells, cancer cells acquire following metabolic alterations: rapid energy generation, enhanced biosynthesis of macromolecules (including carbohydrates, proteins, lipids, and nucleic acids), and maintenance of appropriate cellular redox status. The enhanced aerobic glycolysis (the Warburg effect) not only provides a rapid ATP generation, but also provides a biosynthetic advantage and contributes to a proper control of redox balance for tumor cells, which confers tumor cells advantages of proliferation and survival [16,23-25]**.** Reversing the Warburg effect in tumor cells has been shown to greatly compromise the tumorigenicity of cancer cells [26,27], which suggests that targeting the metabolic changes could be an effective strategy for cancer treatment.

In addition to the Warburg effect, tumors show alterations in many other aspects of metabolism, including altered metabolism of amino acids and lipid. For instance, many tumors are addicted to glutamine, which is a non-essential amino acid for human beings [28-30]. Glutamine can be used for synthesis of proteins and nucleotides, and ATP generation for rapidly growing cancer cells. Glutamine can be converted into glutamate by glutaminase, which can be further converted into α-ketoglutarate, an important substrate for the tricarboxyclic acid (TCA) cycle to produce ATP in cells, a process named glutaminolysis. Furthermore, many tumor cells show high rates of de novo lipid synthesis [31-34]. Lipids have been reported to promote different aspects of cancer development, including promoting the growth, proliferation, and survival of tumor cells, and maintaining redox balance [31].

### Regulation of cancer metabolism by oncogenes and tumor suppressors

Recent studies have begun to reveal underlying molecular mechanisms for the altered metabolism in cancer. The activation of oncogenes (such as Myc, HIF-1α, and PI3K/Akt) and inactivation of tumor suppressors (such as p53, PTEN, TSC2 and LKB1) in cancer cells have been shown to contribute to metabolic alterations in cancer [23-25,35]. Myc promotes glucose uptake through the up-regulation of glucose transporter 1 (GLUT1), and stimulates glycolysis through transcriptional up-regulation of many glycolytic enzymes, including lactate dehydrogenase A (LDHA), hexokinase 2 and phosphofructokinase [36]. In addition to promoting glycolysis, Myc also activates glutaminolysis [30,37]. Myc transcriptionally induces glutamine transporters SLC38A5 and SLC1A5 to promote glutamine uptake in cells. Furthermore, Myc induces the expression of glutaminase 1 (GLS1), an enzyme that converts glutamine into glutamate as the first rate-limiting enzyme of glutaminolysis, by negative regulation of the expression of miR-23a and miR-23b, microRNAs that repress GLS1 expression [37]. HIF-1α activation and stabilization in tumors also contributes greatly to the enhanced glycolysis. HIF-1α stimulates glycolysis through direct transactivation of glucose transporters (such as GLUT1) and many glycolytic enzymes (such as pyruvate dehydrogenase kinase 1 (PDK1) and pyruvate kinase type M2 (PKM2)) [36,38]. PDK1 is a kinase that phsphorylates and inhibits pyruvate dehydrogenase, which converts pyruvate into acetyl-CoA for TCA cycle. The induction of PDK1 by HIF-1α slows the conversion of pyruvate into acetyl-CoA and prevents the entry of pyruvate into the TCA cycle [39,40]. PKM2 is a fetal isoform of pyruvate kinase, which is highly expressed in many tumors. Interestingly, PKM2 is ineffective at promoting glycolysis compared with other pyruvate kinase isoforms, such as PKM1 which is expressed in the muscle and brain [41,42]. By slowing glycolysis, PKM2 results in a buildup of carbohydrate metabolites in cells that can be used to generate macromolecules to support the rapid growth and proliferation of tumor cells. In addition to Myc and HIF-1α, activation of PI3K/AKT signaling pathway also plays a critical role in activating glycolysis. AKT induces the translocation of glucose transporters, GLUT1 and GLUT4, to cell surface to promote glucose uptake [43]. AKT also activates glycolytic enzymes, such as hexokinase 2 and phosphofructokinase 1 and 2. Furthermore, AKT phosphorylates and inactivates tumor suppressor TSC2, a negative regulator of mTOR, to promote glycolysis [44]. The mTOR signaling pathway plays a critical role in tumorigenesis and metabolism [17,45]. Activation of mTOR has been reported to induce HIF-1α, which in turn induces PKM2 expression to promote glycolysis [46].

In addition to the activation of oncogenes, inactivation of tumor suppressors has been recently shown to lead to the metabolic alterations in cancer. For instance, PTEN can inhibit glycolysis as well as glutaminolysis through both PI3K/AKT-dependent and -independent pathways [47]. TSC2 inhibits glycolysis through its negative regulation of mTOR. LKB1 negatively regulates glycolysis through its activation of AMP-activated protein kinase (AMPK), which activates TSC2 to negatively regulate the activity of mTOR [48]. Recent studies have shown that p53 plays an important role in regulation of cellular metabolism, including mitochondrial oxidative phosphorylation, glycolysis, glutaminolysis, and fatty acid oxidation. Considering that p53 is mutated in over 50% of human cancer and dysfunctional in more cancer, loss of p53 function should be an important mechanism contributing to the metabolic alterations in cancer.

### p53 regulates mitochondrial oxidative phosphorylation and glycolysis

**Figure 1:**
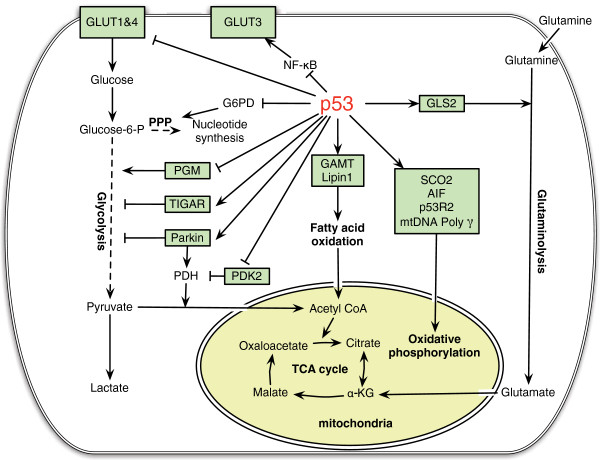
**The regulation of cellular metabolism by p53.** p53 regulates mitochondrial oxidative phosphorylation, glycolysis, glutaminolysis and fatty acid oxidation in cells. p53 transcriptionally induces SCO2, AIF and p53R2, and physically interacts with mtDNA Poly γ to maintain the mitochondrial integrity and promotes oxidative phosphorylation. p53 reduces glucose uptake through direct repression of the transcription of GLUT 1 & 4, and indirect repression of the expression of GLUT 3. p53 negatively regulates PGM at the protein level and transcriptionally induces TIGAR and Parkin to inhibit glycolysis. Parkin positively regulates PDH, which converts pyruvate into acetyl-CoA. p53 negatively regulates the expression of PDK2, which phosphorylates and inhibits the PDH activity. p53 induces the expression of GLS2, which catalyzes the hydrolysis of glutamine to glutamate. The latter can be further converted into α-KG (α-ketoglutarate). By increasing the levels of α-KG, GLS2 promotes TCA cycle and oxidative phosphorylation. p53 physically interacts with G6PD to negatively regulate the activity of G6PD, and thereby down-regulates PPP (pentose phosphate pathway), a pathway critical for nucleotide synthesis and NADPH production. p53 induces the expression of GAMT and Lipin1 to promote fatty acid oxidation. By producing acetyl-CoA, fatty acid oxidation contributes to the maintenance of TCA cycle and mitochondrial oxidative phosphorylation.

p53 plays a critical role in maintaining the integrity of mitochondria and oxidative phosphorylation and down-regulation of glycolysis (Figure 1). Loss of p53 results in the deficiency of mitochondrial oxidative phosphorylation and enhanced glycolysis in both cultured cells and mouse models [14]. Several p53 target genes have been identified to mediate the role of p53 in maintaining mitochondrial oxidative phosphorylation, including SCO2 (synthesis of cytochrome c oxidase 2), AIF (apotosis-inducing factor), GLS2 (glutaminase 2), Parkin and p53R2. The SCO2 is a key regulator of the cytochrome c oxidase complex (mitochondrial complex IV) that is essential for mitochondrial oxidative phosphorylation. p53 induces the expression of SCO2 to ensure the maintenance of the cytochrome c oxidase complex, thereby enhancing oxidative phosphorylation [14]. AIF is required to maintain the integrity of mitochondrial complex I in the mitochondrial electron transport chain. p53 transcriptionally induces AIF to promote mitochondrial oxidative phosphorylation [49,50]. GLS2 is a mitochondrial glutaminase that catalyzes the hydrolysis of glutamine to glutamate. p53 increases GLS2 expression, which enhances the production of glutamate and a-ketoglutarate in cells, and thereby promotes mitochondrial oxidative phosphorylation and ATP generation [51,52]. Parkin was recently reported to contribute to the function of p53 in regulating oxidative phosphorylation [53]. Parkin is a gene associated with Parkinson disease, one of the most common neurodegenerative diseases. Mutations of the Parkin gene account for most autosomal recessive forms of juvenile Parkinson disease. Parkin has been recently shown to be a potential tumor suppressor. Diminished expression and mutations of the Parkin gene have been frequently observed in various tumors [54,55]. Parkin deficiency results in reduced mitochondrial oxidative phosphorylation [53]. Furthermore, Parkin increases the protein expression of pyruvate dehydrogenase E1α1 (PDHA1). PDHA1 is a critical component of the pyruvate dehydrogenase (PDH) complex, which catalyzes the conversion of pyruvate into acetyl-CoA, a primary substrate for TCA cycle, and serves as a critical link between glycolysis and oxidative phosphorylation. Parkin deficiency reduces the levels of PDHA1 protein, leading to the decreased levels of acetyl-CoA, which contributes to the impaired oxidative phosphorylation and enhanced glycolysis in cells [53]. p53 also activates PDH through its transcriptional repression of pyruvate dehydrogenase kinase-2 (PDK2), which inhibits PDH by phosphorylation of PDHA1 [56]. The repression of PDK2 expression by p53 in turn promotes conversion of pyruvate into acetyl-CoA instead of lactate, and promotes the mitochondrial oxidative phosphorylation and represses the glycolysis in cells. Ribonucleotide reductase subunit p53R2 plays an important role in the maintenance of mitochondrial DNA (mtDNA). Loss of p53R2 results in decreased mtDNA and mitochondrial function in cells. Through the transcriptional up-regulation of p53R2, p53 maintains the integrity of mtDNA and mitochondrial oxidative phosphorylation [57,58]. In addition to the transcriptional regulation of its target genes, p53 also maintains mitochondrial genetic stability through its interaction with mtDNA polymerase γ (mtDNA Poly γ) in response to mtDNA damage induced by intracellular and extracellular stress, including ROS. p53 protein physically interacts with mtDNA Poly γ to enhance DNA replication function of Poly γ and therefore mitochondrial function. Loss of p53 results in a significant increase in mtDNA vulnerability to damage, leading to increased frequency of mtDNA mutations [59]. These findings together provide strong evidence that p53 plays a critical role in maintaining mitochondrial integrity and promoting mitochondrial oxidative phosphorylation.

In addition to promoting mitochondrial oxidative phosphorylation, p53 represses aerobic glycolysis through regulating glucose transporters and glycolytic enzymes (Figure 1). p53 reduces glucose uptake through direct repression of the expression of glucose transporters 1 & 4 (GLUT1 and GLUT4) [60], and indirect repression of the expression of glucose transporter 3 (GLUT3) [61]. The latter occurs through the negative regulation of NF-κB signaling by p53. p53 reduces the activity of NF-κB signaling by inhibiting the activities of IκB kinase α and β, which in turn reduces the expression of GLUT3 [61]. p53 also regulates the enzymes involved in glycolysis, such as PGM (phosphoglycerate mutase) and TIGAR (*T*P53-induced glycolysis and apoptosis regulator). PGM acts at the later stage of glycolytic pathway. p53 promotes the ubiquitiation and degradation of PGM protein. Loss of p53 results in the increased PGM expression, thereby enhancing glycolysis [62]. TIGAR is another target of p53 which negatively regulates glycolysis in cells [15]. TIGAR functions as an enzyme that dephosphorylates fructose-2,6-bisphosphate to fructose-6-phosphate. This activity of TIGAR counteracts that of phosphofructokinase, a key regulatory enzyme in glycolysis. By lowering the intracellular levels of fructose-2,6-bisphosphate, TIGAR reduces glycolysis and diverts glucose catabolism to the pentose phosphate pathway (PPP). Parkin also contributes to the function of p53 in negative regulation of glycolysis. Parkin deficiency promotes glycolysis, whereas ectopic expression of Parkin reduces glycolysis in tumor cells [53]. Currently, it is still unclear whether Parkin can directly repress glycolysis or whether the enhanced glycolysis resulted from Parkin deficiency is simply due to the impaired mitochondrial oxidative phosphorylation.

p53 can inhibit the pentose phosphate pathway to further regulates glucose metabolism in cells (Figure 1) [63]. The pentose phosphate pathway is important for both glucose metabolism and biosynthesis in cancer cells. In an oxidative phase, the pentose phosphate pathway generates reduced NADPH, an important intracellular reductant required for reductive biosynthesis such as ribose 5-phosphate, the precursor for biosynthesis of nucleotides. This is followed by a non-oxidative interconversion of ribose 5-phosphate to the intermediates in the glycolytic pathway. p53 protein directly binds to glucose-6-phosphate dehydrogenase (G6PD), the first and rate-limiting enzyme in the pentose phosphate pathway, and prevents the formation of active G6PD dimmer [63]. Through the inhibition of pentose phosphate pathway, p53 suppresses glucose metabolism and biosynthesis. Loss of p53 results in enhanced pentose phosphate pathway, which in turn increases glucose uptake and directs glucose towards biosynthesis in tumor cells.

**Figure 2:**
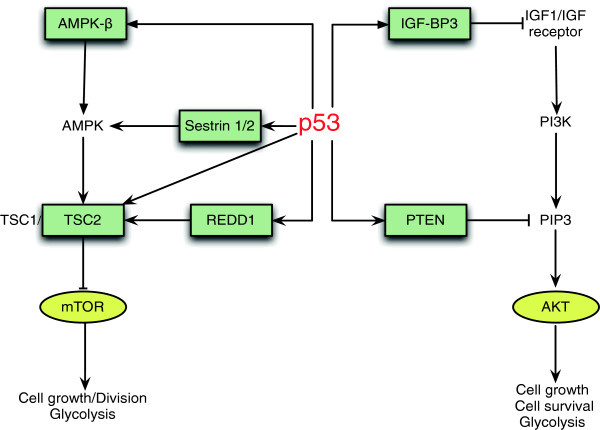
**p53 negatively regulates the PI3K/AKT and mTOR pathways to down-regulate glycolysis.** The PI3K/AKT and mTOR pathways play critical roles in stimulating glycolysis in tumors. p53 negatively regulates the PI3K/AKT signaling through inducing the expression of IGF-BP3 and PTEN. p53 also negatively regulates mTOR activity through inducing the expression of AMPK-β, Sestrins 1/2, TSC2 and REDD1.

p53 can further regulate glycolysis through its regulation of PI3K/AKT and mTOR signaling pathways (Figure 2). The aberrant activation of these two pathways has been frequently observed in various tumors, which plays an important role in stimulating glycolysis and promoting growth and proliferation of tumor cells [24,64,65]. p53 negatively regulates these two pathways through inducing a group of target genes that act as negative regulators of these two pathways [17,66,67]. p53 induces IGF-BP3 (insulin-like growth factor binding protein 3), which binds to IGF1 and prevents its binding to IGF receptor, resulting in the down-regulation of the PI3K/AKT signaling. p53 also induces PTEN to negatively regulate the PI3K/AKT signaling [68,69]. As a lipid phosphatase, PTEN dephosphorylates phosphatidylinositol 3,4,5-trisphosphate (PIP3), the second messenger produced by PI3K, and thereby inhibits the PI3K/AKT signaling pathway. AMPK is a major upstream negative regulator of mTOR. p53 activates AMPK through its induction of AMPK-β and Sestrins 1/2, leading to the down-regulation of the mTOR activity [69,70]. p53 also induces TSC2 to negatively regulate the mTOR activity. Furthermore, p53 induces the expression of REDD1, which inhibits the mTOR activity by releasing TSC2 from the association with inhibitory 14-3-3 proteins in response to hypoxia [69,71,72]. Through the regulation of multiple p53 targets in the PI3K/AKT and AMPK/mTOR pathways, p53 negatively regulates the activities of these two pathways, which in turn leads to inhibition of glycolysis in cells. In summary, through the regulation of many different p53 target genes and several different pathways, p53 promotes mitochondrial oxidative phosphorylation and inhibits aerobic glycolysis, leading to the negative regulation of the Warburg effect.

### p53 regulates glutaminolysis and lipid metabolism

In addition to glucose metabolism, p53 regulates many other aspects of cellular metabolism, including glutaminolysis and fatty acid oxidation (Figure 1). GLS2 acts as a mediator of p53's role in glutaminolysis [51]. p53 increases the expression of GLS2, which in turn increases the production of glutamate and α-ketoglutarate in cells, leading to the enhanced mitochondrial oxidative phosphorylation and ATP generation. At the same time, GLS2 increases the levels of antioxidant glutathione (GSH) in cells through increasing the levels of intracellular glutamate, a precursor for GSH, thereby lowering the ROS levels in cells. It has been reported that GLS2 levels are significantly decreased in liver and brain tumors [51,52,73]. Ectopic expression of GLS2 in tumor cells significantly reduced tumorigenicity [51,52,73]. These results strongly suggest a potential role of GLS2 in tumor suppression. Interestingly, GLS2 shares a considerable degree of sequence similarity with GLS1. It was reported that the expression of GLS1 can be induced by Myc, which in turn promotes the proliferation of lymphoma and prostate tumor cells [37]. It is still unclear why GLS1 and GLS2 have contrasting roles in tumorigenesis, and furthermore, the mechanism for GLS2 in tumor suppression remains unclear [51].

Emerging evidence also shows that p53 is involved in lipid metabolism. In response to nutritional starvation, p53 induces fatty acid oxidation to drive the TCA cycle to provide energy for cells (Figure 1). Guanidinoacetate methyltransferase (GAMT) is a critical enzyme that synthesizes creatine. The creatine-phosphocreatine system plays an essential role in energy storage and transmission by re-synthesizing ATP. This system provides a quick source of energy for cells or tissues with high-energy demands, such as muscle and brain. GAMT was identified as a p53 target gene that plays as a key downstream effector of adaptive response to nutritional stress [74]. In response to glucose starvation, GAMT is induced by p53 activation, which in turn up-regulates fatty acid oxidation. Interestingly, GAMT ablation reduces glucose starvation-induced apoptosis in cells, which suggests that GAMT induction by p53 provides energy for p53-mediated apoptosis in response to glucose starvation. Lipin1 was recently identified as another p53-regualted gene that mediates the role of p53 in fatty acid oxidation [75]. Lipin1 is essential for normal adipose tissue development and is also an important regulator of fatty acid metabolism [76,77]. As a nuclear transcriptional coactivator, Lipin1 interacts with a complex containing peroxisome proliferator-activated receptor (PPAR) α and PPAR γ coactivator-1α (PGC-1α) to regulate the expression of genes involved in fatty acid oxidation. As a phosphatidate phosphatase enzyme, Lipin1 catalyzes the conversion of phosphatidate to diacylglycerol. Lipin1 is induced by p53 in response to glucose starvation, which in turn results in the up-regulation of fatty acid oxidation [75]. Therefore, GAMT and Lipin1 connect p53 to fatty acid oxidation in response to nutritional stress. By converting acyl-CoA to acetyl-CoA, fatty acid oxidation connects to TCA cycle and contributes to the maintenance of mitochondrial oxidative phosphorylation. Therefore, the regulation of fatty acid oxidation by p53 could also contribute to the function of p53 in maintaining mitochondrial oxidative phosphorylation and repressing glycolysis in cells.

### p53 regulates antioxidant defense

**Figure 3:**
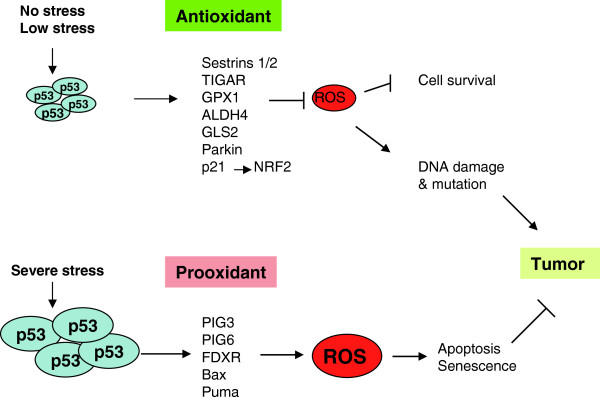
**The regulation of oxidative stress and ROS by p53.** p53 exerts either antioxidant or prooxidant activity depending on extent of stress signals. Under the conditions of nonstress or low stress, p53 induces antioxidant genes, such as sestrins 1/2, TIGAR, GPX1, ALDH4, GLS2 and Parkin, to lower ROS levels in cells. Furthermore, p53 induces p21 to stabilize NRF2, a transcription factor which induces the expression of antioxidant genes to lower ROS levels. This antioxidant activity protects cells from oxidative stress-induced DNA damage and mutations, and also promotes cell survival. Under the conditions of severe stress, p53 induces prooxidant genes, including PIG3, PIG6, FDRX, Bax and Puma, to further induce ROS levels in cells, which in turn further activates p53. This prooxidant activity leads to the p53-mediated apoptosis and senescence to prevent the propagation of mutation-bearing cells. Thus, both antioxidant and prooxidant activities of p53 contribute to the role of p53 in tumor suppression.

Oxidative stress and increased levels of ROS in cells play an important role in tumorigenesis. Recent studies have shown that reducing the ROS levels and enhancing antioxidant defense is an important mechanism of p53 in tumor suppression. Mitochondrial oxidative phosphorylation is a main source of endogenous ROS in cells. Whereas p53 promotes mitochondrial oxidative phosphorylation to maintain the homeostasis of cellular energy metabolism, p53 also plays a fundamental role in reducing the intracellular ROS levels mainly resulted from mitochondrial oxidative phosphorylation and maintaining the redox balance. To exert its antioxidant function, p53 induces a group of antioxidant genes, including sestrins 1/2, TIGAR, GPX1, ALDH4, GLS2, and Parkin, especially under conditions of nonstress or low stress, to lower ROS levels and prevent DNA damage (Figure 3) [15,18,51,53,78,79]. Sestrins are a family of proteins required for regeneration of peroxiredoxins, which are thiol-containing peroxidases and major reductants of peroxides in cells [18]. TIGAR diverts glucose through the pentose phosphate pathway, increasing the levels of antioxidant NADPH [15]. GPX1 is a primary antioxidant enzyme that scavenges hydrogen peroxide or hydroperoxides in cells [78]. ALDH4 is a NAD^+^ dependent enzyme in mitochondrial matrix, which catalyzes proline degradation in mitochondria and thereby reduces intracellular ROS levels [79]. By increasing the intracellular levels of antioxidant GSH, both GLS2 and Parkin can reduce ROS levels in cells [51-53]. In addition to the direct transcription regulation of antioxidant genes, p53 also reduces ROS levels in cells by promoting the stabilization of NRF2 through its up-regulation of p21 [80]. As a transcription factor, NRF2 induces several antioxidant genes, and thus plays a critical role in antioxidant defense in cells. Under nonstressed conditions, NRF2 is constantly ubiquitinated by the Cul3–Keap1 ubiquitin E3 ligase complex and rapidly degraded. In response to oxidative stress, the induction of p21 by p53 inhibits the interaction between NRF2 and Keap1, leading to the increase of NRF2. Consistent with the function of p53 in regulating genes involved in antioxidant defense, p53 deficiency results in the elevation of intracellular ROS levels, which greatly increases DNA oxidation and the rate of mutagenesis in cells. These effects could be substantially reversed by ectopic expression of antioxidant p53 target Sestrins in p53 null cells or by the application of antioxidant N-acetylcysteine both in vitro and in vivo [18,19].

Interestingly, in addition to the function of antioxidant defense, p53 can exert prooxidant function through transcriptional up-regulation of a group of prooxidant genes depending on the levels of oxidative stress that cells are facing (Figure 3). In response to severe oxidative stress, the intracellular levels of ROS are elevated, which activate p53, leading to the p53-mediated apoptosis and senescence to eliminate cells damaged by oxidative stress. At the same time, the activated p53 protein induces the expression of prooxidant genes, including PIG3, PIG6, FDXR, Bax, and Puma, all of which can further increase intracellular ROS levels and promote the p53-mediated apoptosis and senescence to maintain genomic integrity [81-84].

## Conclusions

Here we reviewed our current understanding of the role and mechanisms of p53 in maintaining the homeostasis of cellular energy metabolism and the redox balance in cells. Whereas cell-cycle arrest, apoptosis, and senescence are traditionally accepted as the major mechanisms by which p53 exerts its tumor suppressive function, these findings provide strong evidence that the functions of p53 in maintaining the homeostasis of cellular energy metabolism and the redox balance in cells contribute significantly to the role of p53 as a tumor suppressor.

Metabolic alterations are a hallmark of cancer cells. Ample evidence has shown that the metabolic alterations are critical for the growth, proliferation and survival of tumor cells. Therapeutic strategies targeting cancer metabolism are being developed and tested for cancer treatment. For instance, Metformin, an anti-diabetic drug, which regulates cell metabolism and the AMPK/mTOR signaling, has been already selected as a candidate for tumor therapy [85,86]. Recent studies have shown that reactivation of p53 in tumors leads to the tumor regression in animal models, which provide further evidence that p53 can be targeted for cancer therapy [87,88]. A number of small molecular drugs that activate p53 have been developed, including Nutlins. Nutlins interact with MDM2 to release p53 from the interaction with MDM2, which leads to the p53 activation [89,90]. p53 is emerging as a key regulator of the homeostasis of cellular metabolism; p53 regulates mitochondrial oxidative phosphorylation, glycolysis, glutaminolysis and fatty acid oxidation. However, it is still not fully understood the roles and mechanisms of p53 in regulation of different aspects of cellular metabolism, and how the regulation of these different aspects of cellular metabolism contributes to the function of p53 in tumor suppression. A better understanding of the function of p53 and its target genes involved in the regulation of cellular metabolism could lead to a better understanding of tumorigenesis and identification of new targets for tumor therapy.

## Competing interests

The authors declare that they have no competing interests.

## Authors’ contributions

YL and JL prepared the initial draft of the paper. ZF modified and finalized the paper. All authors read and approved the final manuscript.
